# Acknowledgement to Reviewers of *Non-Coding RNA* in 2017

**DOI:** 10.3390/ncrna4010002

**Published:** 2018-01-23

**Authors:** Non-Coding RNA Editorial Office

**Affiliations:** MDPI AG, St. Alban-Anlage 66, 4052 Basel, Switzerland

Peer review is an essential part in the publication process, ensuring that *Non-Coding RNA* maintains high quality standards for its published papers. In 2017, a total of 24 papers were published in the journal. Thanks to the cooperation of our reviewers, the median time to first decision was 25.50 days and the median time to publication was 62.50 days. The editors would like to express their sincere gratitude to the following reviewers for their time and dedication in 2017:
Alvarez-Dominguez, Juan R.Li, HongdaAniello, FrancescoLi, LinAtianand, Maninjay K.Lin, Chin-YoBacolla, AlbinoLiu, ChangningBazzini, ArielLiu, JunBenistant, ChristineMelters, DanielBiswas, SantanuMelters, DanielBlanco, EnriqueMo, Yin-YuanBlin, KaiMoran, YehuBolger, AnthonyMoreno-Mateos, MiguelBorges, FilipeMueller, SoerenBose, DebojitNecsulea, AnamariaBudak, HikmetNelson, AndrewChakrabarti, KausikNowicki, MarcinChakrabarti, KausikOler, AndrewChiang, Tai-anPanda, AmareshCiaudo, ConstancePathania, RajneeshCock, J. MarkPichler, MartinD'Agnano, IgeaSanchez-Carbayo, MartaDibb, NicholasSchmittgen, ThomasDimri, GoberdhanSingh, KasmirDonato, MicheleSlotkin, KeithDragomir, AndreiStathopoulos, ConstantinosDweep, HarshSterne-Weiler, TimEnguita, FranciscoSutaria, DhruvitkumarFava, EugenioTaguchi, YoshihiroFerracin, ManuelaTesfaye, DawitFerreira, LucianaUchida, ShizukaFiannaca, AntoninoUchida, ShizukaGarcía-Escudero, RamónUnver, TurgayGonzález, José M.Unver, TurgayGupta, Sachin KumarValdmanis, PaulHamamah, SameerVanderWeele, DavidHiroshi, NakayamaVaughan, AshleyJanga, MadhusudhanaVolders, Pieter-JanJanitz, MichaelVolders, Pieter-JanJiang, WeiVora, MehulJin, HailingWang, JiguanJordan, KingWang, LixiaoKav, Nat N. V.Wang., KaiKim, TaewanXiao, JunKosaka, NobuyoshiXuan, ZhenyuKotsikorou, EvangeliaYakovlev, Igor A.Kuchenbauer, FlorianYamada, MasashiKumar, RajeshYu, XiangKumar, Rajive


